# Tuberculous Peritonitis

**Published:** 1897-11-27

**Authors:** E. Percy Paton

**Affiliations:** Surgical Registrar to Westminster Hospital, and Surgeon to the Mildmay Hospital


*(To be continued).*

### TUBERCULOUS PERITONITIS.

By E. Percy Paton, M.D., M.S.Lond., F.R.C.S., Surgical Registrar to Westminster Hospital, and Surgeon to the Mildmay Hospital.

Until recent years peritonitis of tuberculous origin was a disease which came only under the cognisance of the physician, except in the few cases in which some acute trouble, such as intestinal obstruction, supervened in the course of the disease, or suppuration occurred; but since, as it were, almost by an accident, it has been found that laparotomy exercised a beneficial effect, these cases are coming more and more into the surgeon's hands.

Three varieties of the disease are usually described, the main conditions found in each of which will be shortly indicated here, before passing to the clinical features of the condition—(1) The ascitic cases are mainly characterised by the presence of a large quantity of fluid, usually more or less free, in the abdomen, while on the peritoneum in many places smaller or larger tubercular nodules are to be seen. (2) The fibrous variety is thought by some to be merely a stage in the process of cure of the ascitic; but certainly this is not always the case, as sometimes it comes without any preceding accumulation of fluid. Its main feature is the matting together of the abdominal contents by multiple adhesions, amongst which adhesions are many evidences of tubercle, the individual original miliary variety having coalesced into masses which are especially connected with the often shrunken and rolled up omentum. Amongst these many adhesions fluid may collect in places, giving rise to a condition which has many times in women been mistaken for ovarian cyst. (3) In the ulcerative variety, while matting from adhesions takes place, the tuberculous deposit shows a great tendency to break down, and form localised collections of pus and caseous *débris*, which may burst externally or into the intestines (which are often the seat of tuberculous ulceration), but do not usually spread widely in the peritoneal cavity, owing to the numerous adhesions. Tuberculous peritonitis often forms part of a general widespread deposit of tubercle in other organs, especially in the other serous membranes, particularly the pleura; we propose here, however, to deal only with those cases in which the lesion is confined to the peritoneum.

**Symptoms.**—Treves states that the disease is most common between the ages of 20 and 40; all authorities do not, however, agree to this, it being considered by many to be more common in patients under 20. All, however, agree that it is more common in males than females.

The symptoms to which the first onset of the trouble gives rise are often very obscure, being mainly ill-defined abdominal pain and discomfort, often of a colicky nature, accompanied by attacks of diarrhœa, which last for a short time, and then subside. Often the stomach is in an irritable condition, so that indigestion, with occasional attacks of nausea, which may go on to vomiting, occur, for which no cause can be assigned. At the same time the appetite is generally bad or fickle, and the tongue thinly furred, while the face is often pale and pasty. If the temperature be taken it will usually be found to be raised at night; but only a degree or two, being normal again in the morning.

Physical examination at this stage will often reveal little beyond a somewhat tympanitic abdomen, but, as the case goes on, the ordinary signs of fluid will be found, and perhaps masses of irregular shape and somewhat firm to the touch, which may be fairly constant in position, or sometimes freely moveable, may be felt. These are, perhaps, most common in the umbilical region, and are due to the rolled up and matted omentum, which contains masses of tuberculous deposit. In other cases there will be a full abdomen, with irregular patches of dulness, which give other signs of fluid, and are due to loculated collections amongst the adhesions.

Should pus form, which it may do in these cases, even generally in the peritoneum, without causing very acute general symptoms, as it is tuberculous in origin and not septic, redness and bulging of the umbilicus may be present, which may give way, the pus finding exit by this means. Sometimes, however, the pus is loculated amongst adhesions, and the redness appears only over its surface, the skin becomes shiny and thin, and finally gives way, a discharging sinus being formed. In this, as in other tuberculous diseases, the patient gradually wastes during its progress, and the most favourable prognostic sign is any improvement in the general condition.

**The Diagnosis** in many cases is exceedingly easy; but in others it is just as difficult. In those cases where there is a large quantity of fluid it is mostly arrived at by a process of exclusion, there being no heart, lung, liver, or kidney disease to account for the ascites, while at the same time the general condition, with slight rise of temperature and variable digestive troubles, with masses of thickened peritoneum felt on palpation, settle the question. In cases where a distinct mass or masses can be felt, and form the most prominent feature, these may be mistaken for new growth, usually malignant, or for enlarged and moveable spleen or kidney, &c. Careful palpation and watching the case for a time will generally clear up any doubt of this kind. Most mistakes are made because most difficulty is encountered in distinguishing between an encysted collection of fluid due to tubercle and an ovarian cyst. This difficulty is sometimes increased by the fact that tubercle in the female commences in the Fallopian tubes and then spreads to the peritoneum, so that the fluid collection may be apparently or even truly connected with the pelvic organs and in the situation in which an ordinary ovarian cyst would be found. Careful examination, however, will usually clear up the case by revealing the fact that the apparent cyst is not the only abdominal trouble, there being other fluid collections or masses of tuberculous deposit elsewhere, while the main swelling will sometimes not be uniformly dull to percussion, parts being even tympanitic, indicative of the presence of gas containing gut incorporated in it.

